# Comment on the trial protocol “Early versus late BCG vaccination in HIV-1-exposed infants in Uganda: study protocol for a randomized controlled trial”

**DOI:** 10.1186/s13063-019-3213-y

**Published:** 2019-02-12

**Authors:** Sanne Marie Thysen, Ane Bærent Fisker

**Affiliations:** 1 Bandim Health Project, Bissau, Guinea-Bissau; 2 Research Centre for Vitamins and Vaccines, Bandim Health Project, Statens Serum Institut, Copenhagen, Denmark; 3 Center for Global Health (GloHAU), Aarhus University, Aarhus, Denmark; 4 OPEN, Institute of Clinical Research, University of Southern Denmark, Odense, Denmark

We have read with interest the study protocol by Nankabirwa et al. [1]. The authors propose a study of the non-specific effects (NSEs) of Bacillus Calmette-Guérin (BCG) vaccine in HIV-1-exposed children. Children will be randomised to BCG at birth or at 14 weeks of age. Follow-up for the main outcome is planned from 0 to 14 weeks.

As the authors discuss, NSEs of vaccines has been widely debated, and the field has been fraught with much controversy. An important contributor to the controversy is the methodological challenges in analysing observational data, particularly where information on vaccination is incomplete. In 2014, the World Health Organisation (WHO) commissioned a systematic review of the evidence of NSEs. The reviewers concluded that the existing evidence suggested beneficial NSEs of BCG, and that more research, especially randomised controlled trials (RCTs), was needed [2].

A RCT may indeed provide important information. However, in order to settle the controversy it is important that the trial tests the same hypothesis around which the controversy stands. In that respect, we see some problems in relation to the RCT proposed by Nankabirwa et al. The existing literature on NSEs has raised some important issues that should be considered when designing trials to test whether vaccines have NSEs. First, while live vaccines like the BCG vaccine have beneficial NSEs, non-live vaccines like the diphtheria-tetanus-pertussis vaccine (DTP) seem to have negative NSEs. In the WHO review, being DTP-vaccinated was associated with a Mortality Rate Ratio (MRR) of 1.38 (0.92–2.08) compared with being DTP-unvaccinated; restricting the analysis to studies with prospective follow-up and without survival bias, DTP was associated with a two-fold higher mortality (MRR 2.00 (1.50–2.67)) [3]. Second, the most recent vaccine is most important for NSEs: Mortality is lower among BCG-vaccinated children compared with BCG-unvaccinated children [2], whereas among BCG-vaccinated children, DTP vaccination is associated with higher mortality [4]. Therefore, the recent review was based on the estimates with the shortest follow-up period, before administration of subsequent vaccines [2]. Third, NSEs may be sex-differential: in a meta-analysis, DTP-vaccinated girls had a MRR of 2.54 (1.68–3.86) compared with DTP-unvaccinated girls [4], whereas there was no effect for boys (MRR 0.96 (0.55–1.68) [4].

In the present study protocol, Nankabirwa et al. plan a follow-up to 14 weeks. They do not comment on other vaccines administered to the enrolled children during follow-up. In Uganda, DTP-containing vaccine (pentavalent vaccine: diphtheria, tetanus, pertussis, hepatitis B and haemophilus influenza type b vaccines), rota vaccine, pneumococcal vaccine, and oral polio vaccine is recommended at 6 and 10 weeks of age, and thus within the follow-up period. We hypothesise that there will be a beneficial effect of early BCG vaccination among children not yet vaccinated with pentavalent vaccines, but that the beneficial effect will be reduced or even reversed after pentavalent vaccination. Evidence of this has previously been observed in Greenland, Finland and Denmark where BCG at birth was associated with lower infectious disease morbidity until reception of non-live DTP-containing vaccines, but not after DTP [5]. The planned comparison after 6 weeks is BCG-then-DTP versus DTP not preceded by BCG. Thus, the RCT may well end up with a null effect, concealing a beneficial effect from 0 to 6 weeks, but no effect or a tendency for the opposite from 6 to 14 weeks, after pentavalent vaccines are given.

Whether BCG has NSEs in HIV-1-exposed children should, therefore, not be assessed between enrolment and 14 weeks, but between enrolment and 6 weeks. We therefore suggest that Nankawirba et al. in addition to the already planned analyses include an analysis of early versus late BCG vaccination considering only the period from randomisation to 6 weeks of age, when subsequent vaccines are scheduled. According to Fig 2 in the study protocol, a face-to-face interview is conducted at 6 weeks of age and thus the data to conduct the analysis should be available. If feasible, blood samples at 6 weeks may also be informative.

The strongest NSEs of BCG are observed in populations with the highest mortality [6], presumably because infections cause the vast majority of deaths. We would, therefore, expect the present trial to reveal smaller differences in mortality in a setting with close follow-up and prophylactic antibiotics.

The study is very important and with all the resources invested, we think that as much as possible should be learned from the investment. We look forward for the results and hope that Nankabirwa et al. will consider our suggestions.

## References

[1] Nankabirwa V, Tumwine JK, Namugga O (2017). Early versus late BCG vaccination in HIV-1-exposed infants in Uganda: study protocol for a randomized controlled trial. Trials.

[2] Higgins JP, Soares-Weiser K, Lopez-Lopez JA (2016). Association of BCG, DTP, and measles containing vaccines with childhood mortality: systematic review. BMJ.

[3] Aaby P, Ravn H, Benn CS (2016). The WHO review of the possible non-specific effects of diphtheria-tetanus-pertussis vaccine. Pediatr Infect Dis J.

[4] Aaby P, Ravn H, Fisker AB, Rodrigues A, Benn CS (2016). Is diphtheria-tetanus-pertussis (DTP) associated with increased female mortality? A meta-analysis testing the hypotheses of sex-differential non-specific effects of DTP vaccine. Trans R Soc Trop Med Hyg.

[5] Benn CS, Sorup S (2016). Commentary: BCG has no beneficial non-specific effects on Greenland. An answer to the wrong question?. Int J Epidemiol.

[6] Biering-Sørensen S, Aaby P, Lund N (2017). Early BCG-Denmark and neonatal mortality among infants weighing < 2500 g: a randomized controlled trial. Clin Infect Dis.

